# The role of NMDARs in the anesthetic and antidepressant effects of ketamine

**DOI:** 10.1111/cns.14464

**Published:** 2023-09-07

**Authors:** Liang Zhou, Jingjing Duan

**Affiliations:** ^1^ Department of Pharmacology, College of Pharmaceutical Sciences Soochow University Suzhou China; ^2^ Department of Anatomy and Neurobiology, Zhongshan School of Medicine SunYat‐sen University Guangzhou China

**Keywords:** anesthetic effects, antidepressant effects, ketamine, metabolites, NMDARs

## Abstract

**Background:**

As a phencyclidine (PCP) analog, ketamine can generate rapid‐onset and substantial anesthetic effects. Contrary to traditional anesthetics, ketamine is a dissociative anesthetic and can induce loss of consciousness in patients. Recently, the subanaesthetic dose of ketamine was found to produce rapid‐onset and lasting antidepressant effects.

**Aim:**

However, how different concentrations of ketamine can induce diverse actions remains unclear. Furthermore, the molecular mechanisms underlying the NMDAR‐mediated anesthetic and antidepressant effects of ketamine are not fully understood.

**Method:**

In this review, we have introduced ketamine and its metabolism, summarized recent advances in the molecular mechanisms underlying NMDAR inhibition in the anesthetic and antidepressant effects of ketamine, explored the possible functions of NMDAR subunits in the effects of ketamine, and discussed the future directions of ketamine‐based anesthetic and antidepressant drugs.

**Result:**

Both the anesthetic and antidepressant effects of ketamine were thought to be mediated by *N*‐methyl‐d‐aspartate receptor (NMDAR) inhibition.

**Conclusion:**

The roles of NMDARs have been extensively studied in the anaesthetic effects of ketamine. However, the roles of NMDARs in antidepressant effects of ketamine are complicated and controversial.

## Graphical abstract

The NMDAR inhibition is the primary presumptive molecular mechanism underlying the anesthetic and antidepressant effects of ketamine.

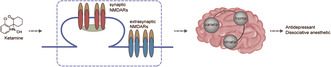

## 1 INTRODUCTION

Over 300 million surgeries are performed annually,^1^, ^2^ and anesthesia is used worldwide to deal with these painful surgeries.^2^ The use of general anesthesia is characterized by amnesia, analgesia, unconsciousness, and immobility.^3^ As a dissociative anesthetic, ketamine produces rapid‐onset effects while retaining consciousness.^4^ However, the psychological side effects of ketamine during the recovery period and the high potential of abuse prevent its clinical application.^5^, ^6^ Recently, a subanaesthetic dose of ketamine was found to generate rapid‐onset and long‐lasting antidepressant effects.^7^, ^8^ Low concentrations of ketamine can induce rapid antidepressant effects within 1 h of a single infusion that last for over 1 week, which has been used for treatment‐resistant depression (TRD).^9^, ^10^ Nonetheless, ketamine has the potential for abuse and psychological side effects, restricting its clinical use for treating depression. Therefore, investigating the molecular mechanisms underlying the anesthetic and antidepressant effects of ketamine would benefit ketamine‐based drug discovery as well as decrease the abuse potential and side effects of ketamine.

Ketamine is an *N*‐methyl‐D‐aspartate receptor (NMDAR) antagonist, and NMDAR inhibition is assumed to mediate the anesthetic and antidepressant effects of ketamine.^11^, ^12^, ^13^ Accordingly, several NMDAR antagonists, such as nitrous oxide (N_2_O, laughing gas) and MK‐801, can produce effects similar to those of ketamine.^11^, ^14^ Although the antidepressant effects of ketamine are sustained for weeks,^15^, ^16^ the half‐life of ketamine is 2–4 h, and it metabolizes rapidly and extensively to norketamine (NK), hydroxynorketamine (HNK), and dehydronorketamine (DHNK).^17^, ^18^ Therefore, the metabolites of ketamine are assumed to mediate its long‐term effects. Moreover, NK can induce anesthetic effects, and HNK can produce antidepressant effects, indicating that the metabolism of ketamine affects its pharmacology. However, in a later study, HNK was found to be inessential for the antidepressant effects of (R)‐ketamine,^19^ suggesting that the molecular mechanisms underlying the ketamine‐induced anesthetic and antidepressant effects need further investigation.

NMDAR inhibition is the primary presumptive molecular mechanism underlying the effects of ketamine.^13^, ^20^ Both ketamine and its metabolite NK bind NMDARs and produce anesthetic effects.^21^ However, the local anesthetic effects of ketamine may also be mediated by voltage‐gated sodium channels.^22^, ^23^ HNK can enhance the activation of alpha‐amino‐3‐hydroxy‐5‐methyl‐4‐isoxazolepropionic acid receptors (AMPARs) and mediate the antidepressant effects of ketamine in an NMDAR‐independent manner.^24^, ^25^, ^26^ By contrast, although HNK can bind NMDARs,^27^ it does not produce antidepressant effects.^19^, ^28^ The presumptive mechanisms underlying NMDAR inhibition in the effects of ketamine are controversial and much more complicated. In this review, we have focused on the NMDAR‐induced anesthetic and antidepressant effects of ketamine. First, we have introduced ketamine and its metabolites. Then, we have summarized the recent advances in the NMDAR‐mediated anesthetic and antidepressant effects of ketamine and have surveyed the possible roles of different NMDAR subunits in the effects of ketamine. Finally, we have discussed the future perspectives on the clinical application of ketamine.

## 2 KETAMINE

Phencyclidine (PCP) was designed and synthesized for use as a dissociative anesthetic in 1958.^29^ However, due to its strong psychomotor, rewarding, and reinforcing properties, PCP and its analogs were abused worldwide as psychoactive substances, although their psychopharmacological properties have not yet been fully uncovered.^30^

Psychomimetic adverse effects were found to have limited the clinical usefulness of PCP. Since then, more PCP analogs have been designed to reduce these psychotomimetic and cognitive adverse effects.^31^ Ketamine (Ci581) (2‐(*O*‐chlorophenyl)‐2‐methylamino cyclohexanone) is one of over 200 analogs of PCP and exhibits promising anesthetic and antidepressant effects.^32^ Ketamine has two optical isomers (enantiomers): *S*‐(+)‐ketamine and *R*‐(−)‐ketamine.^33^ In this review, ketamine has been referred to as a racemic mixture. Ketamine, or “Special K” causes a dissociative state of relaxed well‐being and hallucinogenic effects at subanaesthetic doses.^34^ Moreover, the well‐known adverse psychological effects of ketamine would impair cognition and memory, which have not yet been solved.^35^ As a rapid‐acting drug, the half‐life of ketamine is 2–4 h. Ketamine is catalyzed through N‐demethylation by cytochrome P450 enzymes and can generate the initial metabolite (R, S)‐(NK) (80%) in human plasma.^12^ NK is metabolized into DHNK through dehydrogenation, and ketamine can also be rapidly metabolized into HK and 6‐HNK through hydroxylation and *N*‐demethylation.^36^, ^37^, ^38^ To further understand the metabolism and pharmacokinetics of ketamine, please see the relevant review.^17^

## 3 THE ANESTHETIC EFFECTS OF KETAMINE

The general anesthetic effects of ketamine include amnesia, analgesia, unconsciousness, and immobility.^3^ As a dissociative anesthetic, ketamine causes loss of orthostatic reflexes but not consciousness, indicating that the patients remain awake with their eyes open.^39^, ^40^ As a PCP analog, ketamine exerts anesthetic effects in a dose‐dependent manner by acting on the central nervous system. In 1965, Edward Domino used ketamine as an anesthetic in humans, and the results showed that ketamine was short‐acting with psychotropic effects.^41^ Traditionally, ketamine is administered in a dose of 1–4.5 mg/kg intravenously or 6.5–13 mg/kg intramuscularly to induce anesthetic effects in humans, depending on the patient's age and the desired clinical effects.^34^, ^42^ Ketamine can disrupt frontal–parietal communication and induce anesthetic effects.^43^, ^44^, ^45^, ^46^ Ketamine also affects the cardiovascular system by acting on the sympathetic nervous system. The anesthetic effects of ketamine are dose‐dependent. However, repeated administration can result in ketamine resistance.^47^ *S*‐(+)‐ketamine is more effective and long‐lasting than a racemic mixture of S‐ and R‐enantiomers. Both (*R, S*)‐ketamine and its initial metabolite (*R, S*)‐NK penetrate the blood–brain barrier, thereby producing anesthetic effects. However, the other metabolite (2R, 6R; 2S, 6S)‐HNK can also penetrate the blood–brain barrier but does not induce any anesthetic effects.^48^

Although ketamine can block voltage‐gated sodium channels and induce local anesthetic effects,^22^, ^23^ the general anesthetic effects of ketamine are supposedly mediated by NMDAR inhibition. Accordingly, HNK without any anesthetic effects showed a lower binding affinity for NMDARs compared with ketamine and NK.^26^, ^49^ The anesthetic dose of ketamine caused neuronal apoptosis and cognitive deficits in rodents and rhesus monkeys,^50^, ^51^, ^52^, ^53^ suggesting that the use of ketamine as a dissociative anesthetic should be strictly scrutinized.

## 4 ANTIDEPRESSANT EFFECTS OF KETAMINE

Similar to PCP, the subanaesthetic doses of ketamine produce antidepressant effects. Seven patients with major depression were intravenously administered 0.5 mg/kg ketamine at Yale University in 2000,^7^ the results showed that the depressive symptoms were significantly improved within the next 2 test days. This was the first clinical trial of ketamine for major depression.^7^, ^34^ Some studies also indicated that ketamine could improve bipolar depression and TRD.^54^, ^55^ Patients with TRD do not respond to the currently available antidepressant pharmacological therapies, and this might result in a high risk of suicidal behaviors.^56^ Thus, novel rapid‐response antidepressant drugs are needed for these patients. The previous pilot studies indicated that *R*‐(−)‐ketamine produces rapid and significant effects in the treatment of TRD and bipolar depression.^57^, ^58^ The response rates of patients with bipolar depression and TRD to ketamine at 4 h were >50%.^59^, ^60^ Ketamine can affect the functional connectivity between the cortex and striatum in depressed individuals and thereby produce antidepressant effects,^61^ although some contradictory results have been reported.^62^ Interestingly, patients with depression with dissociative effects were found to have experienced greater improvements in their depressive symptoms upon ketamine treatment,^63^, ^64^ indicating that the dissociative side effects could help predict the antidepressant efficacy of ketamine in these patients. However, subsequent analysis and studies suggested no relationship between the dissociative side effects and the antidepressant effects of ketamine.^10^, ^65^ Furthermore, the dissociative side effects of ketamine are acute and dissipate in 80 min, and the antidepressant effects occur within 110 min and last for 1 week,^66^, ^67^ suggesting that the ketamine‐mediated dissociative side effects and antidepressant effects depend on diverse signaling pathways. The elimination half‐life of ketamine is 2–4 h,^12^, ^68^, ^69^ and the antidepressant effects of ketamine can last for up to 1 week. Therefore, the molecular mechanisms underlying the long‐lasting antidepressant effects of ketamine need further exploration.

Besides, the antidepressant effects of ketamine are dose‐dependent. There is clear evidence of the efficacy of 0.5 and 1.0 mg/kg intravenously administered subanaesthetic doses of ketamine in TRD, whereas 0.1 mg/kg intravenously administered doses of ketamine cannot significantly improve the health of patients with TRD.^70^, ^71^ The molecular mechanisms underlying the dose‐dependent antidepressant effects of ketamine remain elusive.

## 5 THE BLOCKAGE EFFECTS OF KETAMINE ON NMDARs

**FIGURE 1:**
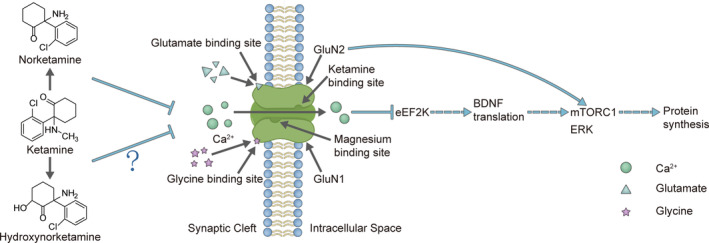
Ketamine and its metabolites inhibit NMDARs. The inhibition of NMDARs by ketamine and its metabolites affects protein synthesis through the actions of BDNF, mTORC1, and ERK.

As a non‐competitive NMDAR antagonist, ketamine can bind the PCP‐binding region of NMDARs in the Ca^2+^ channel pore, resulting in the deactivation of eukaryotic elongation factor 2 (eEF2) kinase (CaMK III), thereby inducing protein synthesis through mTORC1 and executing antidepressant effects (Figure 1).^72^ In line with this, some non‐ketamine NMDAR antagonists such as MK‐801 (dizocilpine), N_2_O (laughing gas) and CPP (3‐(2‐carboxypiperazin‐4‐yl) propyl‐1‐phosponic acid) can also produce fast‐acting antidepressant effects.^11^, ^72^, ^73^, ^74^ However, some non‐ketamine NMDAR antagonists such as memantine, lanicemine (AZD6765), and MK‐0657 (CERC‐301) do not produce robust antidepressant effects.^75^, ^76^ Furthermore, the antidepressant effects of NMDAR antagonists are species‐ and dose‐dependent.^75^, ^76^, ^77^ Taken together, these findings suggested that the roles of NMDARs in ketamine‐mediated antidepressant effects are complicated. Interestingly, the anesthetic doses of ketamine do not induce antidepressant effects, of which the NMDARs are fully blocked by the high concentrations of ketamine.^78^ The subanaesthetic doses of ketamine can partially block the NMDARs, and over 50% of NMDARs are unblocked,^79^, ^80^ which may contribute to the psychiatric side effects of ketamine.

The binding affinity of *S*‐(+)‐ketamine to NMDARs is fourfold greater than that of the other enantiomer *R*‐(−)‐ketamine, indicating that *S*‐(+)‐ketamine has greater potency in anesthetic effects,^81^ although it is more expensive than racemic ketamine.^32^ The psychomimetic side effects of *R*‐(−)‐ketamine were mild as compared with those of *S*‐(+)‐ketamine in depressed mice.^82^ However, the psychomimetic side effects of *S*‐(+)‐ketamine were mostly mild compared with the racemic mixture ketamine in the patients with major TRD.^83^ These inconsistent data from mouse and small‐scale human clinical trials suggest that the psychopharmacological properties of ketamine may be different in different species. Thus, the molecular mechanisms underlying the anesthetic and antidepressant effects of ketamine are complex and need further investigation.

### 5.1 *N*‐methyl‐D‐aspartate receptor

Typically, NMDARs are composed of two GluN1 and two identical GluN2 (2A‐D)/GluN3 (A‐B) subunits and assemble as di‐heteromeric complexes.^84^, ^85^ However, NMDARs can also consist of two GluN1 and two different GluN2/GluN3 subunits, which are tri‐heteromeric complexes.^85^, ^86^ The developmental switch from GluN2B‐ to GluN2A‐containing NMDARs at the hippocampal synapses suggests that NMDAR subunits change during development.^87^, ^88^ All the NMDAR subunits consist of four domains: the NTD (N‐terminal domain) is a clamshell‐like structure, and the ABD (agonist D‐serine/glycine and glutamate‐binding domain) can bind D‐serine/glycine (GluN1 and GluN3) and glutamate (GluN2), the TMD (transmembrane domain) can form the ion channel, and the CTD (C‐Terminal Domain) mediates the synaptic localization of NMDARs and the downstream Ca^2+^ signaling transduction.^89^, ^90^ These agonists are required but are not efficient in the activation of NMDARs because of Mg^2+^ blockage in the NMDARs at rest membrane potential.^91^ Once the postsynaptic membranes are depolarised, Mg^2+^ is released, leading to the opening and activation of NMDARs required for learning and memory.^92^, ^93^, ^94^, ^95^ Ketamine can bind the PCP site in the pore of NMDARs, which is partially overlapped with the Mg^2+^‐binding site.^12^ Therefore, ketamine only blocks the Mg^2+^‐released open NMDARs but not the closed NMDARs with Mg^2+^ blockage.^96^

### 5.2 NMDARs mediated the anesthetic effects of ketamine

Ketamine and its principal metabolite NK are active agents, whereas the other metabolites are inactive compounds.^21^, ^97^ However, the anesthetic effects of NK were significantly decreased as compared with those of ketamine, which indicated that the metabolism of ketamine to NK attenuated the anesthetic effects.^21^ Nevertheless, NK has a lower potential for abuse and side effects than ketamine, especially the S(+) enantiomer.^98^ Interestingly, ketamine's other principal metabolite HNK did not generate any anesthetic effects.^21^ Given that NMDAR inhibition is the major mechanism underlying the anesthetic effects of ketamine, the binding affinity of NMDARs to ketamine (Ki = 530 nM) should be greater than that to NK and HNK. Furthermore, S‐ketamine and its metabolite S‐NK contain five times and eight times higher affinity for NMDARs than the R‐enantiomers, respectively.^99^ Therefore, these S‐enantiomers have more potency in their anesthetic effects compared with the R‐enantiomers. Mechanistically, ketamine binds the PCP site in the NMDAR channel and then inhibits the activation of NMDARs, which is assumed to be the molecular mechanisms through which ketamine induces the anesthetic effects. In line with this, the anesthetic gases xenon and isoflurane can bind the glycine site of NMDARs and inhibit the activation of NMDARs,^100^, ^101^, ^102^ indicating that NMDAR inhibition produces anesthetic effects. In terms of the NMDAR subunits, GluN1 is an obligatory subunit, and is expressed ubiquitously throughout the brain.^85^ The GluN2A and GluN2B subunits are abundant in the central nervous system. However, the GluN2C and GluN2D subunits are restrictedly expressed in the cerebellum.^85^ GluN3 subunits are not required for anesthetic activity.^103^ Therefore, the NMDAR subunits GluN1 and GluN2A‐2B may meet the three criteria of targets relevant to anesthetic action.^104^ Both *GluN1* and *GluN2B* knockout mice die shortly during the postnatal period, so the *GluN2A* knockout mice are the only global knockout mice to prove the essential role of NMDARs in the anesthetic effect of ketamine.^105^ Given that the GluN1, GluN2A, and GluN2B subunits were found to be lost in anti‐NMDA encephalitis,^106^, ^107^, ^108^ such patients should be avoided when using ketamine to induce general anesthesia in the future.^109^

### 5.3 NMDARs were involved in the antidepressant effects of ketamine

The expression of the GluN2A and GluN2B subunits was reduced in the major depression,^110^ the regulation of ellagic acid on the expression of the GluN2A and GluN2B subunits might have affected antidepressant‐like effects.^111^ Taken together, these studies suggested the possible regulation of NMDARs on major depression. The prevailing hypothesis of the antidepressant effects of ketamine was the NMDAR inhibition‐mediated activation of AMPARs (α‐amino‐3‐hydroxy‐5‐methyl‐4‐isoxazolepropionic acid receptors).^112^ Therefore, metabolites with high affinity to NMDARs should have high‐antidepressant potency. However, the antidepressant effects of (R‐)‐ketamine were greater than those of (S‐)‐ketamine,^82^ although the affinity of (R‐)‐ketamine (Ki =1340 nM) to NMDARs was less than that of (S‐)‐ketamine (Ki = 465 nM).^113^, ^114^ In line with this, 50 μM ketamine metabolite (2R, 6R)‐HNK produced more rapid‐acting and reliable antidepressant effects than its enantiomer (2S, 6S)‐HNK.^24^ Furthermore, the NMDAR antagonist MK‐801 (Ki = 3.49 nM) exhibited a higher affinity for NMDARs compared with ketamine (Ki = 530 nM), and MK‐801 did not exhibit more rapid antidepressant effects than ketamine. Taken together, these data suggest that the antidepressant effects of ketamine might be NMDARs‐independent. Furthermore, the ketamine metabolite (2R, 6R; 2S, 6S)‐HNK (Ki > 10,000 nM for NMDARs) could rapidly cross the blood–brain barrier. Given that (2R, 6R; 2S, 6S)‐HNK could generate antidepressant effects, but not anesthetic effects, NMDARs might mainly contribute to the anesthetic effects than to the antidepressant effects of ketamine. Although previous studies have indicated that NMDAR inhibition does not mediate the rapid and sustained antidepressant actions of ketamine,^24^, ^25^ NMDAR inhibition was found to control hyperlocomotion.^24^ Given the confounding factor of hyperlocomotion in the depression mice models,^115^ NMDARs might play a role in the antidepressant effects of ketamine. In line with this, the following study indicated that the 50 μM ketamine metabolite (2R, 6R)‐HNK could inhibit NMDARs, thereby mediating the antidepressant effects of ketamine.^27^ These two inconsistent studies used different concentrations of (2R, 6R)‐HNK to investigate NMDAR inhibition.^24^, ^25^, ^27^ Furthermore, some studies showed that (2R, 6R)‐HNK did not mediate the antidepressant effect of ketamine.^19^, ^116^ These contradicting results highlight the complicated and controversial roles of NMDARs in the anti‐depressant effects of ketamine, and the underlying molecular mechanisms need to be further investigated. Nevertheless, the AMPAR upregulation, brain‐derived neurotrophic factor (BDNF), mammalian target of rapamycin (mTOR) signaling, and protein synthesis contributed to the anti‐depressant effects of ketamine and its metabolite HNK (Figure 1).^24^, ^25^, ^27^, ^78^ Recently, it has been reported that the activation of NMDARs is required for the anti‐depressant effects of ketamine and HNK.^117^ Therefore, the functions of the ketamine metabolite (2R, 6R)‐HNK in NMDAR inhibition and its antidepressant effects are still controversial.^19^, ^28^ The ketamine metabolites (2R, 6R; 2S, 6S)‐HNK did not produce anesthetic effects, which indicated that the anesthetic and antidepressant effects of ketamine were mediated by different metabolites. In line with this, both ketamine and its metabolite NK induced anesthetic effects.^21^ Moreover, if the dissociation side effects were not related to the antidepressant effects of ketamine,^65^ and NMDAR inhibition might not have primarily contributed to these antidepressant effects of ketamine, which further complicated the molecular mechanisms underlying the anesthetic and antidepressant effects of ketamine.

Mechanistically, the activation of AMPARs, ERK–BDNF signaling, mTOR signaling, increased protein synthesis, and increased synaptic density contributed to the long‐lasting antidepressant effects of ketamine.^78^, ^118^, ^119^, ^120^, ^121^ Although both ERK and mTOR signaling regulated protein synthesis, the mTOR signaling might have mediated the antidepressant effects of (S‐)‐ketamine, and the antidepressant effects of (R‐)‐ketamine might have been mediated by ERK signaling (Figure 1).^122^, ^123^ Microglial transforming growth factor‐beta1 was also essential for the antidepressant effects of (R‐)‐ketamine.^124^ Given that the partial blockage of NMDARs by subanaesthetic doses of ketamine induces antidepressant effects, the different subunits of NMDARs may affect the antidepressant effects of ketamine. Accordingly, a previous study has shown that GluN2B selective NMDAR antagonists could induce antidepressant effects,^125^ and that GluN2B‐containing NMDARs mediated the antidepressant effects of ketamine.^119^, ^126^ Furthermore, the GluN2B in γ‐aminobutyric acid (GABA)ergic interneurons, and not the glutamatergic neurons contributed to the antidepressant effects of ketamine,^127^, ^128^ which suggested that the partial blockage of NMDARs induced the antidepressant effects of ketamine.^128^, ^129^, ^130^, ^131^ Mechanistically, GluN2B‐containing NMDAR inhibition by ketamine could activate mTORC1, and subsequently increase the synaptic protein synthesis and spine number, therefore producing rapid antidepressant effects.^78^ However, small‐scale human clinical trials showed that the mTOR inhibitor rapamycin did not block but rather enhanced the antidepressant effects of ketamine,^132^ which suggested that the molecular mechanistic explanation for the antidepressant effects of ketamine in humans and animals should be further investigated.

### 5.4 NMDAR‐mediated excitatory/inhibitory balance contributed to the ketamine activity

**FIGURE 2:**
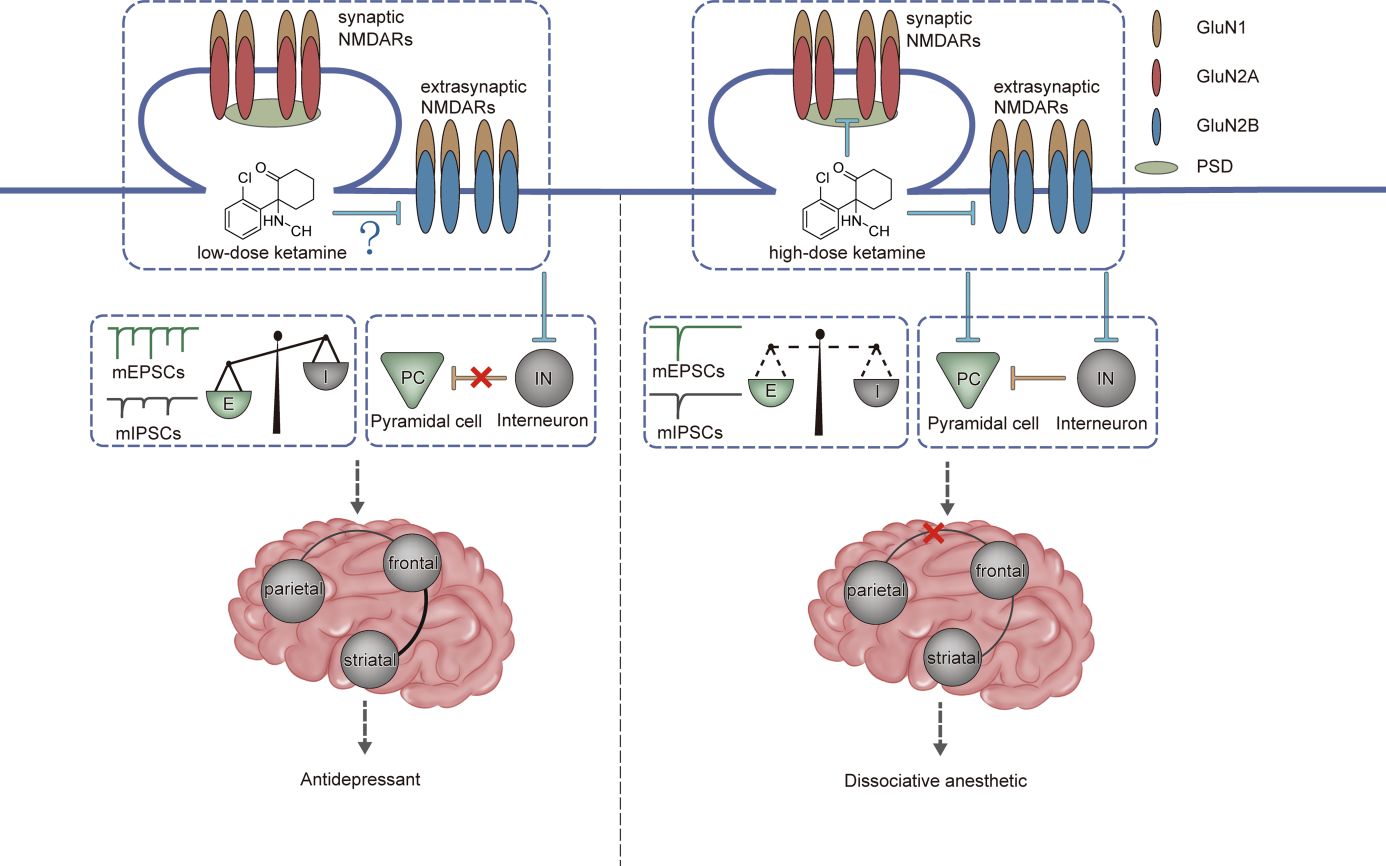
Ketamine and its metabolites produce anesthetic and antidepressant effects by acting on NMDARs. The inhibition of NMDARs by ketamine and its metabolites affects synaptic/extrasynaptic NMDAR‐mediated functions, which might disrupt the balance of excitation and inhibition, thereby generating antidepressant and anesthetic effects.

Typically, primary ionotropic glutamate receptors such as NMDARs and AMPARs, in the glutamatergic excitatory pyramidal neurons generated excitatory synaptic transmissions, and the gamma‐aminobutyric acid type A receptors (GABA_A_Rs) exhibit inhibitory synaptic transmission through GABAergic inhibitory interneurons.^133^ The excitatory/inhibitory (E/I) balance is essential for the processing of cortical information.^134^ The E/I imbalance has been a prominent hypothesis in psychiatric diseases, such as schizophrenia and autism.^135^ Subanaesthetic doses of ketamine could be used to generate a pharmacological animal model of schizophrenia,^136^, ^137^ suggesting that the E/I balance is related to ketamine actions. However, interneurons comprise only 10%–15% of all cortical neurones in rodents, and are required for E/I balance and cortical functions.^138^, ^139^ Pyramidal neurones mediate excitatory synaptic transmission. The interneurons innervate almost every local pyramidal neurone and provide feedforward and feedback inhibition on the excitatory neurones.^140^ Subanaesthetic doses of ketamine blocked the GluN2B‐containing NMDARs in interneurons,^128^ and then reduced the synaptic GABAergic inhibitory synaptic transmission, therefore resulting in the disinhibition of excitatory pyramidal neurons and producing the antidepressant effects.^128^, ^141^ Taken together, these studies demonstrated that the low dose of ketamine shifted the E/I balance towards excitation, thereby reducing the long‐lasting antidepressant effects (Figure 2).

NMDARs can regulate the synaptic localization and functions of AMPARs and GABA_A_Rs,^130^, ^142^ thereby playing crucial roles in E/I balance.^141^, ^143^, ^144^ For more details about the roles of NMDARs in E/I balance, see a recent review.^145^ Low doses of ketamine should also block NMDARs in excitatory pyramidal neurons. However, a decrease in excitatory input would reduce the inhibitory input onto a single pyramidal neuron, generating E/I balance in a cellular autonomous manner.^146^, ^147^ Reversely, the decrease in inhibitory input may not affect the excitatory input.^146^, ^148^, ^149^ Therefore, NMDAR inhibition using low doses of ketamine attenuated the inhibition of interneurons, resulting in E/I imbalance (Figure 2).^150^

The development switch from GluN2B‐ to GluN2A‐containing NMDARs in the synaptic regions and the higher mobility of GluN2B than of GluN2A in the extrasynaptic regions resulted in synaptic localized GluN2A‐containing NMDARs and extrasynaptic localized GluN2B‐containing NMDARs.^151^, ^152^, ^153^ The postsynaptic density consisted of synaptic NMDARs and thousands of proteins,^154^ and exhibited as a dense lamina under an electron microscope.^155^ Therefore, a low dose of ketamine could not freely pass through the postsynaptic density, and could also not block the synaptic NMDARs. Therefore, a low dose of ketamine might only block the GluN2B‐containing NMDARs in extra‐synapses, and a high concentration of ketamine could block the GluN2A‐containing NMDARs in synapses, and the GluN2B‐containing NMDARs in extra‐synapses (Figure 2). Accordingly, animal models of schizophrenia generated using subanaesthetic doses of ketamine,^136^, ^137^ and the hypofunction of NMDARs in interneurons could induce schizophrenia‐relevant phenotypes.^156^, ^157^

## 6 FUTURE PERSPECTIVES

The rapid onset and sustained anesthetic and antidepressant effects of ketamine were dose‐dependent. S‐ketamine induced rapid onset and sustained anesthetic effects without severe psychotomimetic side effects.^158^, ^159^ By contrast, R‐ketamine generated rapid‐onset and long‐lasting antidepressant effects without abuse liability and side effects at a subanaesthetic dose.^82^ Therefore, S‐ketamine might be used as an anesthetic at higher doses, whereas R‐ketamine may exhibit antidepressant effects at lower doses.

Due to the rapid metabolism of ketamine into its downstream metabolites, it is proposed that these metabolites mediate the long‐lasting effects of ketamine. NK could produce anesthetic effects, and HNK could induce antidepressant effects, although these studies were inconsistent and controversial.^19^, ^24^, ^27^ Furthermore, recent studies have shown that these pharmacological properties of the metabolites were significantly attenuated compared to those of unmetabolized ketamine.^21^, ^28^ Therefore, the metabolism of ketamine cannot substantiate the ketamine actions, or unmetabolized ketamine may be responsible for the anesthetic and antidepressant effects, all of which need further investigation.

As an NMDAR antagonist, the roles of NMDARs have been extensively studied in the anesthetic and antidepressant effects of ketamine. However, the roles of NMDARs in the antidepressant effects of ketamine are complicated and controversial. Furthermore, ketamine could also bind/affect other receptors, such as HCN1 channels,^160^, ^161^ σ1 receptor,^162^ dopamine,^163^, ^164^ and serotonin receptors,^165^ although with a lower affinity compared with that of NMDARs, suggesting that other receptor systems might also mediate ketamine‐mediated anesthetic and antidepressant effects. In line with this, HCN1 channels were found to mediate the hypnotic actions of ketamine,^160^, ^161^ and the dopamine receptors affected the antidepressant effects and abuse potential of ketamine.^163^ Moreover, opioid receptors contributed to the antidepressant effects of ketamine.^166^, ^167^ Recent studies have suggested that opioid receptors might mediate the abuse potential of (S‐)‐ketamine.^168^, ^169^ Furthermore, the brain networks such as the connections between the frontal cortex and striatal system, the subgenual anterior cingulate cortex (sgACC), and the posterior cingulate cortex (PCC) system, were abnormal in depressed patients, and this may be restored using low doses of ketamine.^170^ Therefore, investigating the subsequent effects of ketamine on the integrated nervous system would help eliminate side effects.

As the primary molecular targets of ketamine, NMDARs also mediate the analgesic and anti‐inflammatory effects of ketamine, although the underlying molecular signaling pathways remain unclear. Furthermore, the severe side effects of ketamine and its abuse potential are mediated by ketamine‐induced hypofunction of NMDARs.^171^ These would attenuate the clinical applications of anesthetics and antidepressants. Thus, the design and synthesis of neo‐analogs of ketamine with mildly psychoactive effects and a lower abuse potential would benefit patients.

Besides their major ionotropic functions (Ca^2+^ influx), NMDARs also exhibit metabotropic functions, which are Ca^2+^ influx independent.^172^ The metabotropic functions of NMDARs contribute to long‐term depression and the development switch of GluN2A‐ to GluN2B‐containing NMDARs.^173^, ^174^ The contributions of the metabotropic functions of NMDARs to those of ketamine require further investigation.

## CONFLICT OF INTEREST STATEMENT

There were no conflicts of interest.

## Data Availability

Data sharing not applicable to this article as no datasets were generated or analysed during the current study.
